# Case Report: Sintilimab-associated acute interstitial nephritis in a patient with severe thrombocytopenia: clinical diagnosis and treatment strategy when renal biopsy is contraindicated

**DOI:** 10.3389/fmed.2026.1825181

**Published:** 2026-05-26

**Authors:** Zikai Zheng, Junhao Lv

**Affiliations:** 1 Department of Nephrology, Hui'an County Hospital, Quanzhou, China; 2 Department of Nephrology, the First Affiliated Hospital of Zhejiang University School of Medicine, Hangzhou, China

**Keywords:** acute interstitial nephritis, gastric cancer, glucocorticoid, immune-related acute kidney injury, multidisciplinary team collaboration, PD-1 inhibitor, Sintilimab, thrombocytopenia

## Abstract

**Background:**

Immune checkpoint inhibitors (ICIs) have become one of the core drugs for the comprehensive treatment of advanced gastric cancer. As a representative domestic PD-1 inhibitor, sintilimab has shown significant efficacy in clinical application, but the management of immune-related adverse events (irAEs) remains a clinical challenge. Among them, the incidence of immune-related acute kidney injury (ir-AKI) is only 16.5%−17%, but it is mainly manifested as acute interstitial nephritis (AIN), which can progress to irreversible renal failure in severe cases. In particular, when ICIs are combined with potentially nephrotoxic chemotherapeutic drugs (e.g., oxaliplatin), the difficulty of etiological identification increases. Renal biopsy, the gold standard for diagnosis, cannot be performed in the presence of contraindications such as severe thrombocytopenia, further exacerbating the dilemma of diagnosis and treatment.

**Case Summary:**

This paper reports a 68-year-old male patient with gastric antrum adenocarcinoma (cT4aN2M0, stage III) who developed progressive elevation of serum creatinine (from a baseline of 89 μmol/L to 663 μmol/L) accompanied by scattered erythematous papules all over the body, severe thrombocytopenia (minimum 24 × 10^9^/L), renal anemia and other multisystem irAEs after 3 cycles of neoadjuvant therapy with sintilimab combined with oxaliplatin-based regimen. Due to the absolute contraindication of renal biopsy caused by severe thrombocytopenia, clinically presumed sintilimab-associated AIN was diagnosed by comprehensively analyzing the medication timeline, clinical phenotype, laboratory examinations and imaging results, and excluding other causes of renal injury. An individualized glucocorticoid therapy (methylprednisolone 80 mg bid intravenous infusion for 3 days → 80 mg qd → 50 mg qd gradual tapering) was adopted, combined with symptomatic supportive treatment. The patient's renal function recovered significantly (serum creatinine decreased to 182 μmol/L), platelet count returned to the normal range (184 × 10^9^/L), skin rash resolved completely, and the patient was discharged in a stable condition.

**Conclusion:**

Sintilimab-associated AIN can present with the coexistence of multisystem irAEs. In the presence of contraindications to renal biopsy, comprehensive assessment based on medication timeline, clinical phenotype, laboratory indicators and treatment response is the core of diagnosis. Early initiation of individualized glucocorticoid therapy can effectively reverse renal function injury, and multidisciplinary team (MDT) collaboration is the key to optimizing the management of complex irAEs. This case provides a feasible diagnosis and treatment pathway for similar clinical dilemmas.

## 1 Introduction

Immune checkpoint inhibitors (ICIs) represented by PD-1/PD-L1 inhibitors have completely changed the treatment pattern of advanced gastric cancer and significantly improved the survival prognosis of some patients (1). As an independently developed PD-1 inhibitor in China, sintilimab specifically blocks the binding of PD-1 to PD-L1/PD-L2 to abrogate immune escape of tumor cells, and has been approved for the first-line treatment of advanced gastric cancer (2). However, excessive activation of the immune system mediated by ICIs can trigger immune-related adverse events (irAEs) in multiple systemic organs, which have highly heterogeneous clinical manifestations, and some reactions progress rapidly and can be life-threatening in severe cases (3).

The kidney is an uncommon target organ for irAEs (4), among which acute interstitial nephritis (AIN) is the most common pathological type, accounting for about 82.8%-93.3% of ICI-associated renal injury. Renal needle biopsy is the gold standard for the diagnosis of AIN, which can clarify pathological characteristics and exclude other etiologies (e.g., drug-induced tubular necrosis, autoimmune nephropathy) (5). However, in clinical practice, the implementation rate of renal biopsy is low due to contraindications such as coagulation dysfunction, thrombocytopenia, and hemodynamic instability in patients, and less than 30% of ICI-associated nephritis cases are confirmed by biopsy in the real world (6), leading to diagnosis relying on clinical inference and great challenges in treatment decision-making. In addition, the potential nephrotoxicity of chemotherapeutic drugs further increases the complexity of etiological identification when ICIs are combined with chemotherapy (e.g., oxaliplatin) (7).

At present, there are few case reports on multisystem irAEs (kidney, skin, blood) occurring after sintilimab combined with chemotherapy with concurrent contraindications to renal biopsy, and its diagnostic strategies and individualized treatment regimens still need further exploration. This paper reports such a special case, and in combination with the latest literature, deeply analyzes its clinical characteristics, diagnostic difficulties and treatment strategies, so as to provide a reference for clinicians in managing similar cases.

## 2 Case presentation

### 2.1 Clinical data

A 68-year-old male patient was admitted to the Department of Nephrology of our hospital on October 7, 2025 due to “diagnosis of gastric cancer for more than 2 months and progressive elevation of serum creatinine for more than 1 month”. The patient presented to the hospital in July 2025 with “epigastric dull pain accompanied by anorexia”. A painless gastroscopy showed a huge ulcer in the gastric antrum (about 3.5 cm in diameter), reflux esophagitis (LA-B grade), chronic atrophic gastritis (type C3) with bile reflux; pathological biopsy suggested adenocarcinoma (moderately differentiated, partially signet ring cell carcinoma) of the lesser curvature of the gastric antrum. Further gastric MR plain scan + diffusion + enhancement (3.0T) showed gastric cancer in the gastric antrum, invading the full thickness of the gastric wall, with perigastric lymph node metastasis (N2) and no distant metastasis, and the imaging stage was cT4aN2M0, stage III.

The patient had no history of chronic kidney disease, hypertension, diabetes mellitus or autoimmune diseases, with normal baseline renal function (serum creatinine 89 μmol/L, urea 5.2 mmol/L on July 20, 2025).

The patient received 3 cycles of neoadjuvant therapy in the Department of Oncology from August 5 to September 17, 2025:

- Cycle 1 (August 5): Oxaliplatin 220 mg (intravenous infusion, d1) + Sintilimab 200 mg (intravenous infusion, d1);
- Cycle 2 (August 27): The same regimen as Cycle 1;
- Cycle 3 (September 17): Oxaliplatin 220 mg (d1) + Sintilimab 200 mg (d1) + Trilaciclib 300 mg (intravenous infusion, d1, administered before chemotherapy to prevent myelosuppression) + Tegafur Gimeracil Oteracil Potassium Capsules 60 mg (oral, d1-d14, twice daily).
- Concomitant medications during treatment included: the gastric mucosal protective agent teprenone, and anti-emetic agents. No NSAIDs, herbal supplements, or iodinated contrast media were used.

During the treatment, the patient's renal function deteriorated progressively: serum creatinine was 92 μmol/L (normal range) at 1 week after the first cycle of chemotherapy; serum creatinine increased to 185 μmol/L at 2 weeks after the second cycle of chemotherapy; serum creatinine further rose to 356 μmol/L at 1 week after the third cycle of chemotherapy, accompanied by decreased urine output compared to baseline (about 800–1,000 ml per day), anorexia and scattered erythematous papules all over the body (with obvious pruritus). Outpatient review on October 5, 2025: serum creatinine 519 μmol/L, cystatin C 1.88 mg/L, platelet count 47 × 10^9^/L; emergency review on October 7: serum creatinine rose sharply to 663 μmol/L, urea 21.59 mmol/L, uric acid 440 μmol/L. The patient was admitted to our department for further diagnosis and treatment.

### 2.2 Physical examination

Vital signs: Body temperature 36.6 °C, pulse 64 beats/min, respiration 18 breaths/min, blood pressure 133/86 mmHg, weight 79 kg, height 172 cm.

**Figure 1:**
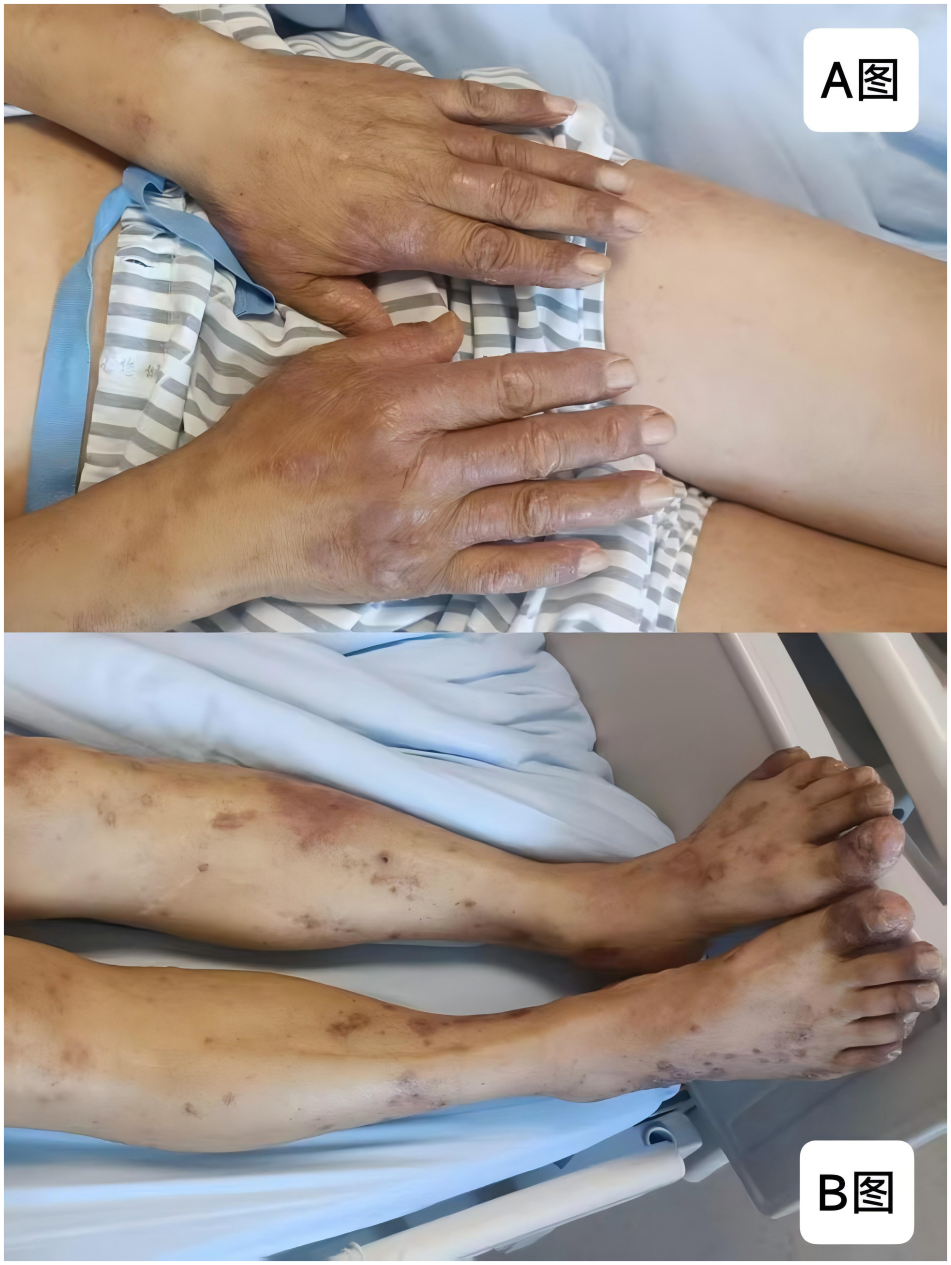
Cutaneous rash of the patient. **(A)** Scattered erythematous papules on both hands with partial fusion; **(B)** Erythematous patches with mild desquamation on both lower extremities.

The patient was conscious with anemic appearance, no jaundice of skin and mucous membranes all over the body, scattered erythematous papules mainly on both hands and lower extremities, some of which fused into patches with mild desquamation and scratch marks (Figure 1); clear breath sounds of both lungs without rales; regular heart rhythm with a heart rate of 64 beats/min; soft and flat abdomen without tenderness or rebound tenderness; no hepatosplenomegaly under the costal margin; no edema of both lower extremities; no other abnormal findings.

### 2.3 Admission laboratory and imaging examinations

#### 2.3.1 Renal function

Serum creatinine 663 μmol/L, urea 21.59 mmol/L, uric acid 440 μmol/L.

#### 2.3.2 Complete blood count

Platelets 35 × 10^9^/L, hemoglobin 81 g/L, white blood cell count 4.52 × 10^9^/L, eosinophil proportion 9.7%.

#### 2.3.3 Urine tests

Urinalysis showed negative occult blood, negative protein, positive glucose; urinary protein-creatinine ratio was 0.07–0.09 g/g, indicating no nephrotic-range proteinuria; mild elevation of urinary β2-microglobulin and retinol-binding protein (RBP) suggested tubulointerstitial injury. No urinary white blood cell casts or red blood cell casts were observed, and urinary eosinophil testing is not routinely performed in our institution.

#### 2.3.4 Immunological evidence

**Figure 2:**
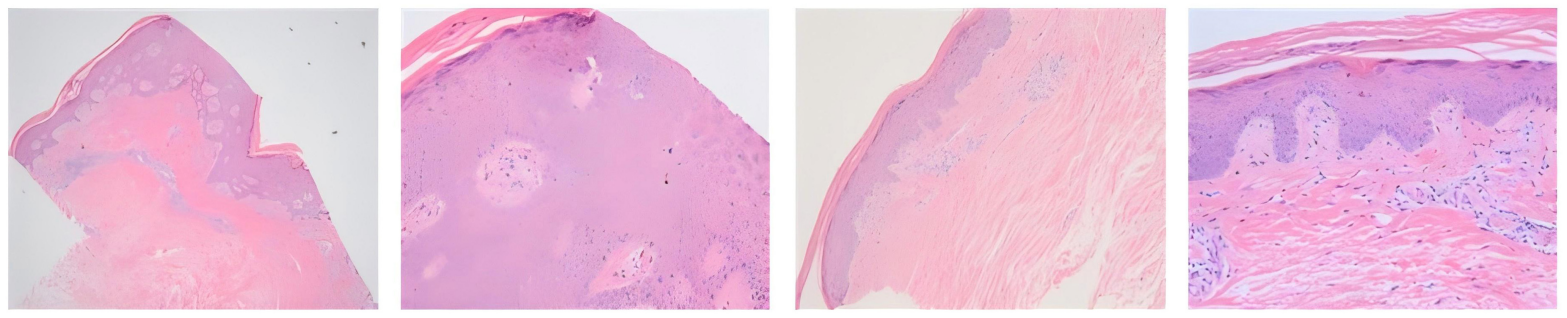
Pathological biopsy result of the patient's skin. Skin biopsy showed hyperkeratosis with focal parakeratosis, scattered dyskeratotic cells in the epidermis, and liquefactive degeneration of basal cells. Perivascular infiltration of inflammatory cells mainly composed of lymphocytes in the superficial and middle dermis, and a large number of melanophages scattered in the superficial dermis.

Total IgE was significantly elevated to 950.25 KU/L (normal < 100 KU/L); eosinophil proportion was 9.7%; skin biopsy confirmed drug-induced dermatitis (Figure 2). Bone marrow aspiration ruled out primary hematological diseases, and mild impairment of megakaryocytic platelet production supported immune thrombocytopenia.

#### 2.3.5 Exclusionary tests

Antinuclear antibody series, anti-neutrophil cytoplasmic antibodies (MPO, PR3), anti-glomerular basement membrane antibody, anti-phospholipase A2 receptor antibody were all negative; renal ultrasound and whole abdominal CT showed no post-renal obstruction; schistocytes accounted for 0.1%−0.2%, ruling out thrombotic microangiopathy; no signs of infection.

#### 2.3.6 Other indicators

Elevated tumor marker CA19-9 (358.7 U/ml); no obvious abnormality in thyroid function (Table 1).

**Table 1:** Core immunological and tumor marker indicators of the patient.

Detection item	Detection result	Normal reference range
Total IgE	950.25 KU/L	0–100 KU/L
Antinuclear antibody series	Negative	Negative
Anti-neutrophil cytoplasmic antibody (MPO)	1.36 RU/ml	0–5 RU/ml
Anti-neutrophil cytoplasmic antibody (PR3)	1.52 RU/ml	0–5 RU/ml
Anti-glomerular basement membrane antibody	1.27 RU/ml	0–5 RU/ml
Carbohydrate antigen 19–9	358.7 U/ml	0–37 U/ml
Carcinoembryonic antigen	4.9 ng/ml	0–5 ng/ml

### 2.4 Diagnosis reasoning

The patient had an absolute contraindication to renal needle biopsy due to the minimum platelet count of 24 × 10^9^/L (extremely high bleeding risk), so a strategy of “exclusion method + comprehensive clinical inference” was adopted to confirm the diagnosis, with specific differentiation as follows:

### 2.5 Key evidence supporting sintilimab-associated AIN

#### 2.5.1 Strict correlation with medication timeline

The patient had normal baseline renal function, and the elevation of serum creatinine had a clear dose accumulation effect with sintilimab administration: normal after Cycle 1 (92 μmol/L) → mild elevation after Cycle 2 (185 μmol/L) → exponential elevation after Cycle 3 (356 → 519 → 663 μmol/L), which was consistent with the pathological characteristic of “cumulative immune activation” of ICI-associated irAEs (8). According to the sintilimab package insert, the incidence of immune-related nephritis is 0.4%, with the first onset time of 32–511 days after medication, and the onset time in this case (63 days after medication) is consistent with the literature report (9).

#### 2.5.2 Characteristic of multisystem irAEs coexistence

Concurrent occurrence of cutaneous irAEs (erythematous papules, hand-foot syndrome), hematological irAEs (severe thrombocytopenia, mild anemia, elevated eosinophils and serum IgE) constituted the “kidney-skin-blood” triad, which was in line with the pathological mechanism of “systemic immune storm” mediated by PD-1 inhibitors. Literature reports that about 44.3% of patients with ICI-associated renal injury are complicated with irAEs in other organs, among which the skin and hematological systems are more common (10), and multisystem involvement can be an important clue for the diagnosis of ICI-associated AIN.

#### 2.5.3 Typical clinical phenotype

Manifested as progressive oliguria and acute renal failure, no hematuria in urinalysis, mostly non-nephrotic-range proteinuria (11) (urinary protein-creatinine ratio 0.09 g/g), mild elevation of urinary β2-microglobulin and RBP, which was highly consistent with the clinical phenotype of ICI-associated AIN reported in the literature. The pathological characteristic of ICI-associated AIN is renal interstitial infiltration of lymphocytes (mainly T lymphocytes), which may be accompanied by plasma cells and eosinophils, and occasional granuloma formation (12), rather than severe glomerular or tubular injury, so there is usually no massive proteinuria or hematuria, which is a key differential point from chemotherapeutic drug-induced nephrotoxicity.

#### 2.5.4 Comprehensive exclusion of other etiologies

- **Exclusion of obstructive nephropathy**: No pelvicalyceal dilatation was found in renal ultrasound and whole abdominal CT, with no evidence of urinary tract obstruction;
- **Exclusion of prerenal renal injury**: The patient had no history of dehydration or hypotension, blood pressure was maintained at about 133/86 mmHg, and urine output did not increase significantly after fluid replacement, which was inconsistent with renal injury caused by volume depletion;
- **Exclusion of thrombotic microangiopathy (TMA)**: Abnormal red blood cell morphology examination showed schistocyte proportion of 0.1%−0.2% (normal range) with no evidence of hemolysis, ruling out oxaliplatin-associated TMA (13);
- **Exclusion of autoimmune nephropathy**: Antinuclear antibody, ANCA, anti-glomerular basement membrane antibody and others were negative, with no evidence of autoimmune diseases;
- **Exclusion of infection-associated renal injury**: The patient had no fever or infection focus, no significant elevation of white blood cell count and neutrophil proportion in routine blood test, and no obvious increase in inflammatory markers (high-sensitivity C-reactive protein), ruling out infectious factors.

### 2.6 Key evidence for excluding oxaliplatin-induced nephrotoxicity

#### 2.6.1 Inconsistent injury pattern

Oxaliplatin-induced nephrotoxicity is mainly manifested as acute tubular necrosis (ATN) or Fanconi syndrome, clinically often accompanied by severe electrolyte disorders (e.g., hypocalcemia, hypokalemia, hypophosphatemia), decreased urine specific gravity and massive proteinuria (14, 15). However, The patient did not have significant electrolyte disturbances and had negative proteinuria, which was inconsistent with the typical manifestations of oxaliplatin-induced nephrotoxicity.

#### 2.6.2 Mismatched onset timeline

Platinum-based chemotherapeutic drug-induced nephrotoxicity mostly occurs 3–5 days after medication with an “acute onset”, while renal injury in this case occurred delayed after the 2nd-3rd cycles and progressed progressively, which cannot be explained by the “acute cumulative toxicity” of chemotherapeutic drugs.

#### 2.6.3 Absence of accompanying neurotoxicity symptoms

The common adverse reaction of oxaliplatin is peripheral neuropathy (16) with an incidence of about 80%, moreover, the patient did not exhibit any symptoms such as limb numbness or paresthesia, which further lowers the possibility of oxaliplatin as the principal pathogenic factor.

### 2.7 Clinical diagnosis conclusion

Based on the above evidence, the final main clinical diagnoses were:

1. Clinically presumed Sintilimab-associated acute interstitial nephritis (immune-related acute kidney injury, CTCAE grade 4);
2. Malignant gastric tumor (cT4aN2M0, stage III);
3. Immune-related thrombocytopenia (CTCAE grade 4);
4. Immune-related eczematous dermatitis (CTCAE grade 2);
5. Secondary hyperglycemia (CTCAE grade 1);

## 3 Treatment and outcome

### 3.1 Treatment REGIMEN

Multidisciplinary team (MDT) collaboration was initiated immediately after admission, and the treatment plan was jointly formulated by the Department of Nephrology, Oncology, Hematology and Dermatology:

#### 3.1.1 Discontinuation of pathogenic drugs

Immediate discontinuation of sintilimab and oxaliplatin, suspension of gastric cancer neoadjuvant therapy to avoid further aggravation of immune injury;

#### 3.1.2 Glucocorticoid therapy

**Figure 3:**
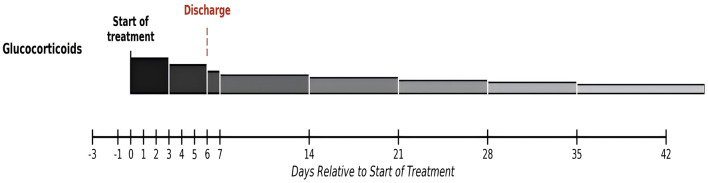
Glucocorticoid tapering protocol. Protocol showing methylprednisolone dosing schedule: 80 mg bid IV × 3 days → 80 mg qd IV × 4 days → 50 mg qd PO → gradual taper by 5 mg/week.

Considering the patient's history of gastric cancer (risk of gastrointestinal bleeding), high-dose glucocorticoid pulse therapy was not adopted. According to the *Chinese Expert Consensus on the Management of Renal Immune-Related Adverse Events Caused by Immune Checkpoint Inhibitors (2025 Edition)* (17), individualized glucocorticoid therapy was given: with a body weight of 79 kg, the patient received moderate-dose methylprednisolone 80 mg bid intravenous infusion for 3 days (loading dose) → converted to methylprednisolone 80 mg qd (1 mg/kg·d) intravenous infusion for 4 days → gradually tapered to 50 mg qd oral administration (Figure 3), which took into account the treatment of renal injury and cutaneous irAEs.

#### 3.1.3 Symptomatic supportive treatment

- **Anti-allergic therapy**: Ebastine Tablets 10 mg qn orally, Halometasone and Triclosan Cream for thin application on the rash area;
- **Other symptomatic support**: Sodium bicarbonate for urine alkalization, atorvastatin for lipid regulation, insulin for blood glucose control, Teprenone for gastric protection, Promote platelet production and correct anemia;
- **Maintenance of water and electrolyte balance**: Monitoring of serum potassium and calcium, timely correction of electrolyte disorders.

### 3.2 Treatment outcome

**Figure 4:**
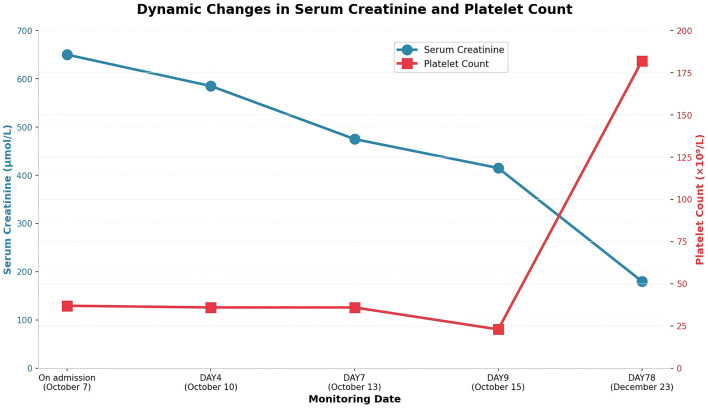
Trends in serum creatinine and platelet count over time. Line graph showing the inverse relationship between serum creatinine (decreasing from 663 to 182 μmol/L) and platelet count (increasing from 35 to 184 × 10^9^/L) over the monitoring period from October 7 to December 23.

The patient's condition improved significantly after treatment:

- **Renal function**: Serum creatinine was 663 μmol/L on admission on October 7, and decreased stepwise after glucocorticoid treatment, to 587 μmol/L on October 10, 477 μmol/L on October 13, 417 μmol/L on October 15; and dropped to 182 μmol/L at 2.5 months after discharge (December 23) (Table 2, Figure 4), urine output recovered to 1,500–2,000 ml/d;
- **Hematological system**: platelet count improved synchronously, rising from 35 × 10^9^/L on admission to 184 × 10^9^/L at 2.5 months after discharge, with the lowest value of 24 × 10^9^/L appearing in the early stage of treatment, hemoglobin increased to 80 g/L, eosinophil proportion returned to normal;
- **Cutaneous symptoms**: General rash basically resolved completely, pruritus symptoms disappeared;
- **Other indicators**: Blood glucose and lipid were well controlled, appetite improved, defecation returned to normal.

**Table 2:** Dynamic changes of core indicators of renal function and routine blood test in the patient.

Monitoring date	Serum creatinine (μmol/L)	Urea (mmol/L)	Uric acid (μmol/L)	Platelet count ( × 10^9^/L)	Hemoglobin (g/L)	White blood cell count ( × 10^9^/L)
On admission (October 7)	663	21.59	440	35	81	4.52
DAY 4 (October 10)	587	26.86	304	34	84	4.81
DAY 7 (October 13)	477	32.39	227	34	86	3.94
DAY 9 (October 15)	417	35.91	225	24	82	4.09
DAY 78 (December 23)	182	12.19	372	184	80	6.51

The patient was discharged in a stable condition after hospitalization, and continued to take glucocorticoid 50 mg qd orally after discharge, with a tapering of 5 mg per week, and regular follow-up in the Department of Nephrology, Oncology and Hematology.

## 4 Discussion

Immunotherapy is a major breakthrough in the field of tumor treatment following surgery, radiotherapy, chemotherapy and targeted therapy. ICIs are a new type of immunotherapeutic drugs. While stimulating anti-tumor immune response, ICIs can cause non-specific activation of T cells, thus breaking the original immune balance of the body. Overactivated immune cells not only attack tumors but also may injure normal tissues, triggering immune-related injuries in multiple systemic organs. Among them, the skin, gastrointestinal tract, endocrine glands, liver and lung are more common, while the heart and kidney are relatively rare. Recent real-world studies have shown that the overall incidence of immune checkpoint inhibitor-associated acute kidney injury (ICI-AKI) is about 16.5%−17% (25). With the increasingly widespread application of these drugs, new research results indicate that the incidence of renal adverse events induced by these drugs may have been underestimated in previous studies.

The immunological mechanism of renal irAEs is mainly as follows: (1) By inhibiting the CTLA-4 and PD-1/PD-L1 signaling axes, ICIs alleviate the suppressive effect on T cell activation and proliferation. This leads to the weakened function of regulatory T cells that maintain self-tolerance, resulting in abnormal activation and proliferation of naive T cells against self-antigens, and the comprehensive breakdown of immune tolerance of the body to its own tissues. (2) Activated autoreactive T cells differentiate into effector T cells and migrate to the kidney. They directly kill renal tubular epithelial cells expressing corresponding antigens (e.g., PD-L1) by releasing toxic substances such as perforin and granzyme, causing direct cellular damage. (3) Infiltrating effector T cells and other activated immune cells secrete a large number of proinflammatory cytokines (e.g., IL-6, IFN-γ, CXCL10, etc.), forming a strong inflammatory microenvironment in the kidney. This “cytokine storm” not only aggravates renal tubular and interstitial injury but also can involve glomeruli, causing podocyte injury. (4) ICIs can also abnormally activate B cells, leading to the production of autoantibodies against renal tissues. These autoantibodies can bind to antigens on renal tubules, mesangium or podocytes to form immune complexes, mediating another type of immune injury through complement activation and other pathways, constituting an auxiliary attack in addition to cellular immunity. Notably, clinical studies have found that renal irAEs are more likely to occur in patients who concurrently use drugs that can easily induce interstitial nephritis such as proton pump inhibitors or non-steroidal anti-inflammatory drugs (17).

The core highlight of this case is that the patient developed multisystem irAEs after sintilimab combined with chemotherapy, could not undergo renal needle biopsy due to severe thrombocytopenia, was clinically presumed to have sintilimab-associated AIN based on comprehensive clinical evaluation, and achieved good curative effect with individualized glucocorticoid therapy. Combined with the existing literature, we discuss the diagnostic difficulties, treatment strategies and clinical implications from three aspects.

### 4.1 Diagnostic dilemmas and solutions

Renal needle biopsy is the gold standard for the diagnosis of AIN, but it could not be performed in this case due to severe thrombocytopenia (24 × 10^9^/L), which is a common dilemma in ICI-associated renal injury in clinical practice (18). At this time, it is necessary to establish a “clinical diagnostic criteria” to replace pathological diagnosis. Referring to the 2025 Chinese Expert Consensus (17), the clinical diagnosis of ICI-associated AIN needs to meet the following conditions: (1). Acute kidney injury occurs after medication (serum creatinine elevation ≥26.5 μmol/L within 48 h, or ≥50% increase compared with baseline); (2). Other definite causes of renal injury (obstruction, dehydration, infection, autoimmune disease, etc.) are excluded; (3). Any one of the following is present: elevated urinary β2-microglobulin or RBP; increased urinary eosinophils; complicated with irAEs in other organs; rapid improvement of renal function after glucocorticoid therapy. This case fully met the above criteria and had the strong prompt signal of “multisystem irAEs”, which further improved the credibility of clinical diagnosis. In addition, the significant elevation of total IgE (950.25 KU/L) also provided evidence for immune-mediated injury. Literature reports that patients with ICI-associated cutaneous irAEs are often accompanied by elevated IgE (19), and although its correlation with renal injury has not been clarified, it can be used as an indirect evidence of immune activation.

### 4.2 Individualized selection of treatment strategies

Glucocorticoids are the first-line treatment for ICI-associated AIN. The 2024 position statement of the American Society of Onconephrology (ASON) recommends that glucocorticoid therapy should be initiated as early as possible once ICI-AKI is suspected, without waiting for biopsy results, because early initiation of treatment is associated with more than a twofold increase in the likelihood of renal function recovery (20). For grade 3–4 renal injury, methylprednisolone 1–2 mg/kg·d can be given by intravenous infusion (21), and immunosuppressants (e.g., mycophenolate mofetil, infliximab) can be added if ineffective (22). As suggested in the 2024 ASON position statement, daily intravenous pulse therapy with methylprednisolone (0.5–1 g) is a viable option for patients with grade 3 ICI-associated AKI (20). However, the patient had a history of gastric cancer, and high-dose glucocorticoids may increase the risk of gastrointestinal bleeding. Therefore, the conventional pulse dose (500–1,000 mg/d) was not adopted, but an individualized regimen of moderate-dose loading (80 mg bid × 3 days) followed by gradual tapering was selected, which not only ensured the therapeutic effect but also reduced the risk of adverse reactions.

Multidisciplinary team (MDT) collaboration played a key role in the treatment process: the Department of Nephrology led the formulation of renal function protection and glucocorticoid therapy plans, the Department of Oncology evaluated the safety of suspending tumor treatment, the Department of Hematology guided thrombopoietic therapy, and the Department of Dermatology assisted in the management of cutaneous irAEs. Literature reports that the MDT model can significantly improve the diagnostic accuracy and treatment efficiency of ICI-associated irAEs, especially for complex cases with multisystem involvement (23).

In addition, bone marrow aspiration showed no abnormal lymphoma or leukemia cells, and no evidence of hemolytic anemia or iron deficiency anemia. The thrombocytopenia in this case was considered to be immune-related (ITP) rather than chemotherapy-induced myelosuppression, which was supported by the mild impairment of megakaryocytic platelet production shown by bone marrow aspiration. The rapid rise of platelet count after glucocorticoid therapy further confirmed that it was mediated by the same immune pathological mechanism as renal injury and cutaneous injury, suggesting that glucocorticoids have extensive anti-inflammatory and immunosuppressive effects and can inhibit the activity of a variety of inflammatory cells and cytokines for ICI-associated multisystem irAEs (24). Therefore, when the same immune pathological mechanism involves multiple organ systems at the same time (e.g., thrombocytopenia, renal injury, cutaneous injury), systemic glucocorticoid therapy can produce inhibitory effects on multiple targets simultaneously.

### 4.3 Dilemma in re-challenging ICIs

Considering the patient's history of severe multisystem irAEs and according to the *Chinese Expert Consensus on the Management of Renal Immune-Related Adverse Events Caused by Immune Checkpoint Inhibitors (2025 Edition)* (17), the patient was classified as grade 4 renal irAE, and re-challenging sintilimab therapy was abandoned. According to the MDT consultation opinion, elective surgical treatment was recommended after the improvement of renal function.

### 4.4 Literature review and clinical significance

Up to now, relatively few cases of sintilimab-associated AIN have been reported at home and abroad, among which even fewer cases are complicated with severe thrombocytopenia. Compared with previous cases, the unique features of this case are: multisystem irAEs involving three major organs (kidney, skin, blood) at the same time; an individualized moderate-dose glucocorticoid regimen was adopted for treatment, avoiding the adverse reactions of high-dose glucocorticoids; significant reversal of renal function after treatment, avoiding the need for hemodialysis in the patient with a good prognosis.

Through the analysis of this case and relevant literature, we draw the following clinical implications:

#### 4.4.1 Strengthen medication monitoring

Enhanced pharmacovigilance is recommended for ICI-treated patients, with renal function and urinalysis assessed prior to each administration and 1–2 weeks post-treatment. Especially for patients receiving combined chemotherapy, with underlying kidney disease or elderly patients, the monitoring frequency should be increased to detect signs of renal injury early.

#### 4.4.2 Pay attention to the correlation of multisystem irAEs

When patients receiving ICI therapy develop irAEs in a single organ, the possibility of involvement of other organs should be alert, and a comprehensive assessment of renal function, routine blood test, skin and other indicators should be performed to avoid missed diagnosis.

#### 4.4.3 Reasonably deal with contraindications to renal biopsy

For patients who cannot undergo renal needle biopsy, clinical inference can be made using the “clinical diagnostic criteria”, and empirical glucocorticoid therapy should be initiated early after excluding other etiologies to avoid delaying the condition.

#### 4.4.4 Individualized treatment and long-term follow-up

The glucocorticoid dose and tapering speed should be adjusted individually according to the patient's underlying diseases and the severity of irAEs. After treatment, long-term follow-up of renal function, immune function and tumor progression should be performed to evaluate the feasibility of ICI re-challenge.

## 5 Conclusion

Sintilimab-associated acute interstitial nephritis has a low incidence but can progress to severe acute renal failure, and it is easy to be confused with other nephrotoxicities when combined with chemotherapy. For patients with contraindications to renal biopsy, comprehensive assessment based on medication timeline, characteristics of multisystem irAEs, laboratory indicators and treatment response is the core of diagnosis. Early initiation of individualized glucocorticoid therapy and multidisciplinary team collaboration management can effectively reverse renal function injury and improve the prognosis of patients. This case provides valuable diagnosis and treatment experience for clinical management of similar complex cases, and also emphasizes the importance of safety monitoring and standardized management of irAEs during ICI therapy.

## Data Availability

The original contributions presented in the study are included in the article/supplementary material, further inquiries can be directed to the corresponding author.
